# The role of orexin and MCH neurons in the hypothalamus in sleep-wake regulation and learning-forgetting balance

**DOI:** 10.3389/fnins.2025.1590556

**Published:** 2025-09-09

**Authors:** Penghong Li, Jing Zhu, Huijuan Zhang, Guorui Ma, Xintong Li, Yuxin Ding, Xuefei Hou, Xin Li

**Affiliations:** Yunan Key Laboratory of Breast Cancer Precision Medicine, Institute of Biomedical Engineering, Kunming Medical University, Kunming, China

**Keywords:** MCH neurons, orexin neurons, sleep-wake, learning-forgetting, hypothalamus

## Abstract

Orexin (Orx) and melanin-concentrating hormone (MCH) neurons in the lateral hypothalamic area are central to regulating the sleep-wake cycle and coordinating memory consolidation and forgetting through dynamic homeostatic mechanisms. This review systematically examines the functional characteristics of these two neural systems and their interactions: first, MCH neurons facilitate the transition from non-rapid eye movement (NREM) to rapid eye movement (REM) sleep at night via a dual regulatory mechanism and regulate the active forgetting of memories via hippocampal neural circuits; second, orexin neurons maintain homeostasis in daytime wakefulness via monoaminergic and cholinergic pathways, while acting as stabilizers during REM sleep stages and enhance memory encoding through amygdala-prefrontal projections. Notably, these two systems show unique antagonistic synergetic dual-mode regulation under the framework of circadian rhythm: orexin neurons maintain the steady state of sleep-wake cycle by promoting wakefulness and inhibiting REM sleep and MCH neurons form dynamic antagonism by inducing sleep and enhancing REM sleep in the sleep-wake dimension, whereas a coordinated balance of information filtering is achieved in the memory regulation dimension through phasic encoding of hippocampal theta rhythms. Clinically, orexin receptor agonists demonstrate therapeutic potential in narcolepsy management, whereas MCH receptor antagonists show promise for memory reconsolidation in post-traumatic stress disorder (PTSD). This review emphasizes the dynamic interplay and reciprocal inhibition between orexin and MCH neurons that form a pivotal bidirectional network framework for dissecting neuropsychiatric comorbidities, wherein pathway dysregulation may propagate from circadian disruption to mnemonic dysfunction, which provides a new theoretical framework for developing intervention strategies across symptom dimensions.

## Introduction

The neuromodulation of the sleep-wake cycle is a complex multi-system integration process involving the synergistic action of multiple brain regions. From a neuroelectrophysiological point of view, the awakening state is characterized by low-voltage fast activity on the electroencephalogram (EEG), a phenomenon that results from the combined action of multiple neurotransmitter systems, including the brainstem, the hypothalamus, and the basal forebrain, on the thalamo-cortical pathway (Nauta, 1946). In contrast, the sleep state stems from the inhibition of the arousal-promoting network by the GABAergic (GABA, gamma-aminobutyric acid) neuronal system and the accumulation of homeostatic sleep factors, such as adenosine and nitric oxide, which ultimately manifests itself as high-amplitude slow-wave oscillations on the EEG (Saper et al., 2010; Jones, 2011; Brown et al., 2012). Disturbance of this fundamental biological process leads to a wide range of adverse physiological consequences. Since the pioneering studies of Von Economo and Nauta (Szymusiak and McGinty, 1986), researchers have demonstrated the critical role of regions of the brainstem, hypothalamus, and basal forebrain in sleep-wake regulation through a variety of methods, including injury experiments, electrical stimulation, and pharmacological interventions (Duque et al., 2000; Lee et al., 2004; Hassani et al., 2009a; Takahashi et al., 2009). Evolutionary biology studies have shown that the neural mechanisms regulating arousal and non-rapid eye movement (NREM) sleep are highly conserved among species, which further highlights their importance in maintaining normal brain function.

Memory formation and consolidation are closely related to the sleep-wake cycle. Information acquired during wakefulness undergoes selective processing during sleep, a process that involves two opposite but equally important mechanisms, memory consolidation and active forgetting (Crick and Mitchison, 1983; Stickgold and Walker, 2013; Feld and Born, 2017). Studies have shown that forgetting is an active process achieved through synaptic renormalization rather than simple memory extinction (Diering et al., 2017; Li et al., 2017). However, there are still many unanswered questions about the specific sleep stage (NREM or REM) in which forgetting occurs and its neural mechanisms (Payne et al., 2008).

**Figure 1:**
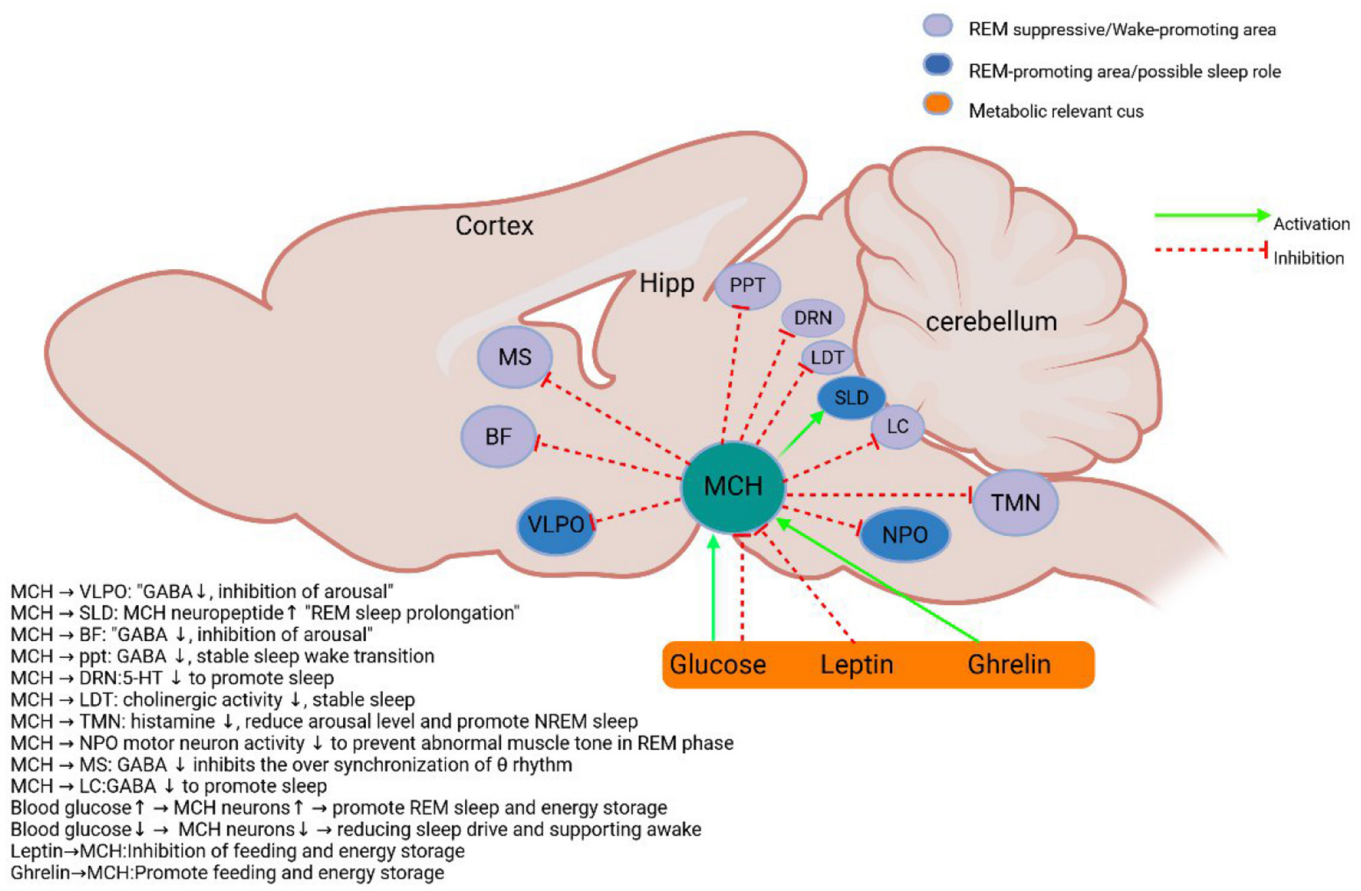
Schematic diagram of MCH system. MCH neurons in the lateral hypothalamus and the initiation zone project to metabolic-related and sleep-wake control nuclei (Verret et al., 2003; Burdakov et al., 2005; Nahon, 2006; Hassani et al., 2009b; Torterolo et al., 2009; Benedetto et al., 2013; Jego and Adamantidis, 2013; Monti et al., 2016). BF, Basal forebrain; DRN, Dorsal raphe nucleus; Hipp, hippocampus; ME, median eminence; PPT, pedunculopontine tegmental nucleus; LDT, Dorsal tegmental; LC, Locus coeruleus; TMN, tuberomammillary nucleus; vLPAG/LPT, Ventrolateral gray matter around the midbrain aqueduct and lateral tegmentum of pons; VLPO, ventrolateral preoptic area; MS, Medial septum; SLD/NPO, Sublaterodorsal tegmental nucleus and pontine nucleus; MCH, Melanin-concentrating hormone neurons. Sleep and metabolism-related nuclei are color-coded, and excitatory and inhibitory inputs are indicated by arrows.

**Figure 2:**
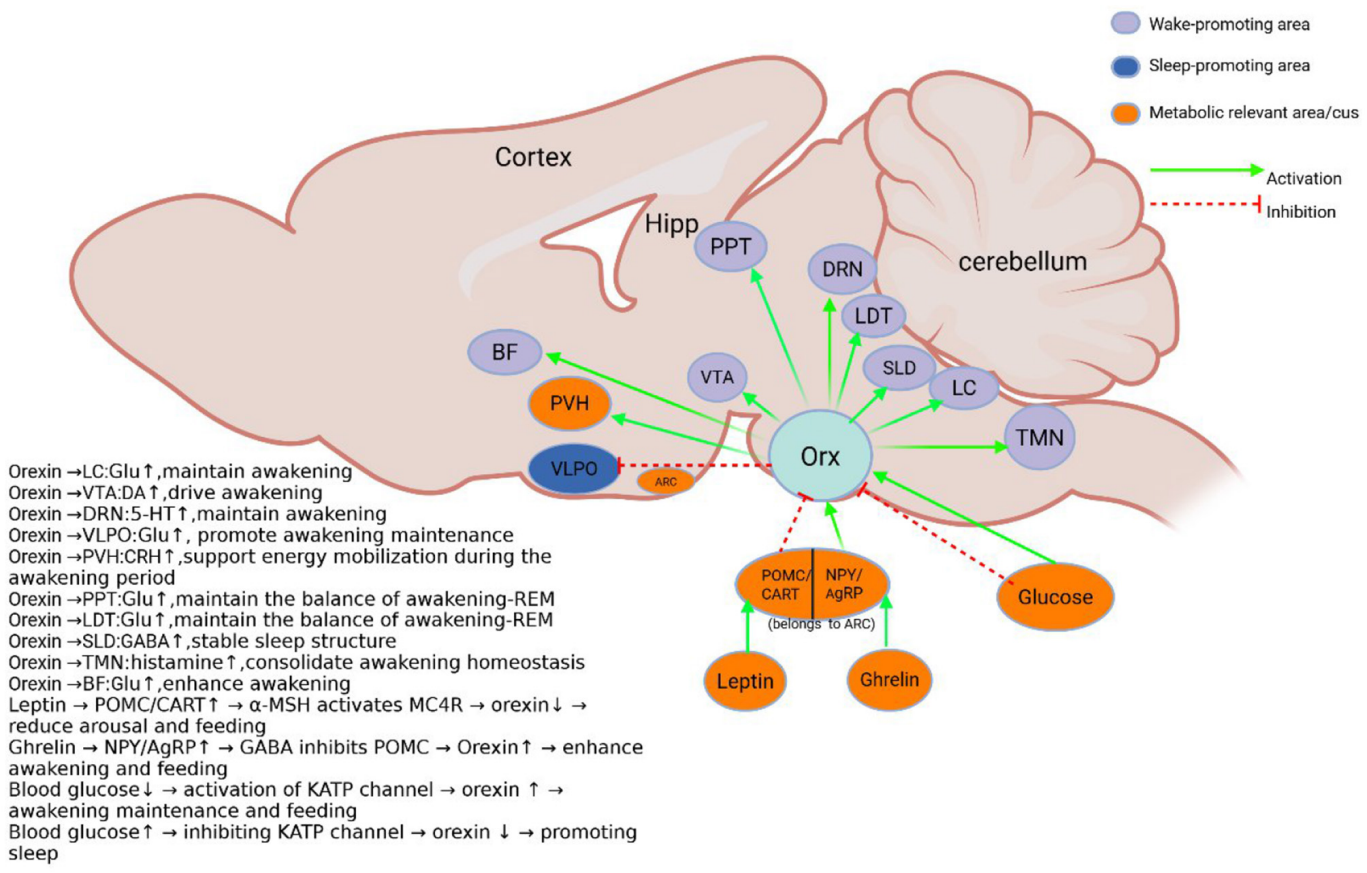
Schematic diagram of the orexin system. Orexin neurons in the lateral hypothalamus project and receive metabolically related projections. And the sleep-wake control nuclei (de Lecea et al., 1998; Peyron et al., 1998; Sakurai et al., 1998; Eggermann et al., 2001; Eriksson et al., 2001; Toshinai et al., 2003; Yamanaka et al., 2003; Burdakov et al., 2006; Chowdhury et al., 2019). BF, Basal forebrain; DRN, Dorsal raphe Nucleus; Hipp, hippocampus; Orx, Orexin; LDT, Dorsal tegmental; PPT, pedunculopontine tegmental nucleus; PVH, Hypothalamic paraventricular nucleus; SLD, Sublaterodorsal tegmental nucleus; LC, Locus coeruleus; TMN, tuberomammillary nucleus; VLPO, ventrolateral preoptic area; VTA, Ventral tegmental area; ARC, Arcuate nucleus; NPY, Neuropeptide Y; AgRP, Agouti-related protein; POMC, pro-opiomelanocortin; CART, Amphetamine related transcripts. Sleep and metabolism-related nuclei are color-coded, and excitatory and inhibitory inputs are indicated by arrows.

The lateral hypothalamic area (LHA), as a key brain region integrating multiple physiological functions, contains two important neuronal populations: orexin (hypothalamic secretin) neurons and melanin-concentrating hormone (MCH) neurons. These two types of neurons have a wide range of central projections (Bittencourt et al., 1992; Peyron et al., 1998; Nambu et al., 1999) (Figures 1, 2) and are involved in the regulation of several physiological processes such as the sleep-wake cycle, memory-memory homeostasis, feeding behavior, and energy metabolism (Sakurai et al., 1998; Shimada et al., 1998; Chemelli et al., 1999; Verret et al., 2003). Among them, orexin neurons play a key role in maintaining arousal homeostasis. Numerous studies have shown that orexin knockout or neuronal ablation results in a narcolepsy-like phenotype (Hara et al., 2001; Adamantidis et al., 2007; Alexandre et al., 2013; Tabuchi et al., 2014), which is highly consistent with the pathological features of human narcolepsy, including excessive daytime sleepiness, hallucinations, and sudden collapse (Burgess and Scammell, 2012; Mahoney et al., 2019). Optogenetic studies have further demonstrated that activation of orexin neurons induces sleep to wakefulness transition (Adamantidis et al., 2007; Tsunematsu et al., 2011; Williams et al., 2019). In addition, the orexin system is involved in the modulation of a variety of memory processes, including situational memory, fear memory, and learning memory (Akbari et al., 2006; Yang et al., 2013). MCH neurons were initially found to be involved in skin pigmentation regulation in fish (Kawauchi et al., 1983), and in mammals they are mainly localized in the LHA. Studies have shown that MCH neurons play an important role in REM sleep regulation: their activation increases REM sleep duration, while inhibition decreases REM sleep (Jego and Adamantidis, 2013; Jego et al., 2013; Konadhode et al., 2013; Tsunematsu et al., 2014) (summarized in Table 1). Notably, recent studies have found that MCH neurons may be involved in memory elimination processes by modulating hippocampal plasticity (Arrigoni et al., 2019; Izawa et al., 2019), a finding that expands our understanding of the function of the MCH system (Yoon and Lee, 2013).

**Table 1:** Summary of studies that investigated the role of the MCH system in sleep-wake regulation.

**Experiment**		**Effect on wakefulness**	**Effect on NREM sleep**	**Effect on REM sleep**	**References**
Microinjection of MCH	DRN	Decreased in wake amounts	Moderate increase in the NREM amounts	Increased in REM amounts Increased in the number of REM bouts No change in the duration of REM bouts	Lagos et al., 2009
	MnR	Decreased in wake amounts	No significant change in NREM amounts	Increased in REM amounts Increased in the number of REM bouts No change in the duration of REM bouts	Pascovich et al., 2021
	LC	No significant change in wake amounts	No significant change in NREM Amounts	Increased in REM amounts Increased in the number of REM bouts No change in the duration of REM bouts	Monti et al., 2015
	BF	Decreased in wake amounts during the first 2-h post-injection	No significant change in NREM amounts	Increased in REM amounts during the first 2-h post-injection Increased in the number of REM bouts No change in the duration of REM bouts	Lagos et al., 2012
	NPO	Decreased in wake amounts during the first hour post-injection	No significant change in NREM amounts	Increased in REM amounts during the first hour post-injection No change in the number of REM bouts No change in the duration of REM bouts Decreased in the latency to REM	Torterolo et al., 2009
	SLD	No significant changes in wake amounts	No significant change in NREM amounts	Decreased in REM amounts during the first and the second 2-h post-injection Decreased in the number of REM bouts No change in the duration of REM bouts increased in the latency to REM	Monti et al., 2016
	VLPO	Decreased in wake amounts during 45h block post-injection Decreased in the duration of Wake bouts	Increased in NREM amounts	No change in REM amounts	Benedetto et al., 2013
MCH l-receptors deletion		No change in wake parameters	No change in NREM parameters	Increased in REM amounts during light period Increased in the number of REM bouts during the light period	Adamantidis et al., 2008
		Increased in wake amounts Increased in the duration of wake bouts No change in the number of wake bouts Increased in wake amounts under restraint stress condition	Decreased in NREM amounts Decreased in the duration of NREM bouts No change in the number of NREM bouts Decreased in NREM amounts under restraint stress condition	No change in REM parameters Decreased in REM amounts under restraint stress condition	Ahnaou et al., 2011
MCH gene knockout		Increased in wake amounts Increased in the duration of wake bouts Increased in wake amounts under fasting condition during both light and dark phase Increased in the duration of wake bouts during both light and dark phase	Decreased in NREM amounts Decreased in NREM amounts under fasting condition during both light and dark phase	Decreased in REM amounts Massive decrease in REM amounts under fasting condition during both light and dark phase Decreased in the duration of REM bouts under fasting condition during both lightened dark phase	Willie et al., 2008
Optogenetic manipulation of MCH	Acute activation of MCH at the onset of NREM		No change in the duration of NREM bouts Increased in the transition from NREM-to-REM		Jego et al., 2013
	Acute activation of MCH at the onset of REM			Increased in the duration of REM bouts	
	Inhibition of MCH at the onset of REM		No changes	Decreased in the frequency and amplitude of REM theta power	
	Acute activation of MCH	No change in wake amounts Increased in the number of wake bouts	Decreased in NREM amounts Decreased in the duration of NREM bouts Increased in the number of NREM bouts increased in the transition from NREM-to-REM	Increased in REM amounts Increased in the number of REM bouts	Tsunematsu et al., 2014
	Acute inhibition of MCH	No changes	No changes	No changes	
Chronic activation of MCH neurons		Decreased in wake amounts during dark phase Decreased in the duration of wake bouts	Increased in NREM amounts during dark phase No change in the duration of NREM bouts Increased in NREM delta power	Increased in REM amounts during dark phase No change in the duration of REM bouts No change in REM theta power	Konadhode et al., 2013
		Deceased in wake amounts during dark phase Deceased in the number of wake long bouts (>32 min)during both day and night phases	Increased in NRE amounts during dark phase Increased in the number of NREM short bouts during both day and night phases Increased in NREM delta power during day phase	Increased in REM amounts during both dark and day phases Increased in the number of REM short bouts during both day and night phases Increased in REM theta power during both night and day phases	Blanco-Centurion et al., 2016
Pharmacogenetic manipulation of MCH	Chemo activation (0.3mg/Kg CNO)	No changes	No changes	Increased in REM amounts during both day and night phases Increased in the number of REM bouts during both day and night phases No change in the duration of REM bouts	Vetrivelan et al., 2016
	Chemo activation (0.5mg/Kg CNO)	–	Deceased in NREM amounts Decreased in the duration of NREM bouts	Increased in REM amounts Increased in the duration of REM bouts	Varin et al., 2018
	Chemo inhibition (5g/Kg CNO)	—	Increased in NREM amounts Increased in the duration of NREM bouts	Decreased in REM amounts No change in the duration of REM bouts	
MCH neurons ablation		Decreased in the duration of wake bouts during the day phase	No changes	Increased in REM amounts Increased in the number of REM bouts during the day phase	Vetrivelan et al., 2016
		Increased in wake amounts during both night and dark phases No change in the duration of wake bouts during both light and dark phases	Decreased in NREM amounts during both light and dark phases Decreased in the duration of NREM bouts during the dark phase No change on EEG power during NREM	No change in REM amounts in both light and dark phases No change on EEG power during REM	Tsunematsu et al., 2014
		No change in wake amounts	No change in NREM amounts	Increase in REM amounts only during the light phase	Varin et al., 2016

Taken together, orexin and MCH neurons in the lateral hypothalamic area play a central role in integrating the sleep-wake cycle and the learning-forgetting balance through a complex network of interactions. These findings not only deepen our understanding of fundamental neurophysiological processes, but also provide new ideas for the treatment of related neuropsychiatric disorders.

## MCH neurons are involved in NREM and REM and hippocampus-dependent memory formation and forgetting

### Mechanistic studies of MCH neurons in sleep regulation: from optogenetics to neural circuit resolution

The application of optogenetic techniques has provided an important method for resolving the role of MCH neurons in sleep regulation. Studies have shown that MCH neurons and orexin neurons are spatially co-localized in the lateral hypothalamic area and both have similar projection target areas (Blanco-Centurion et al., 2016) (Figure 1). This anatomical feature suggests that both may play synergistic or antagonistic roles in sleep-wake regulation. Activation of MCH neurons by optogenetic techniques significantly promotes sleep. Jego et al. (2013) found that optogenetic activation of MCH neurons during wakefulness in wild-type mice induced sleep. This finding was subsequently confirmed by several research teams: While optogenetic activation of MCH neurons consistently increases NREM sleep across studies (Jego et al., 2013; Konadhode et al., 2013), genetic ablation experiments report mixed NREM phenotypes: Tsunematsu et al. (2014) observed decreased NREM after near-complete MCH neuron loss (97%), whereas Varin et al. (2016) found reduced NREM intensity with partial ablation (30%). This divergence likely reflects methodological differences: chronic ablation may trigger compensatory adaptations, whereas acute activation reveals MCH's direct sleep-promoting role. Critically, the dose-dependent effect observed by Varin et al. suggests MCH neurons fine-tune NREM depth rather than simply gate its occurrence. To verify the generalizability of this phenomenon in mammals, Konadhode et al. (2013) used the rAAV-MCH-ChR2(H134R)-EYFP viral vector to specifically express the photosensitive channel, Channelrhodopsin-2 (ChR2), in rat MCH neurons. The experimental results showed that activation of MCH neurons in rodents all promoted sleep by decreasing awakening time and increasing δ-wave power, and this cross-species consistency confirmed the conserved function of MCH neurons in mammalian sleep regulation.

Notably, MCH neurons may have different mechanisms for the regulation of NREM and REM sleep. Tsunematsu et al. (2014) selectively ablated ~97% of MCH neurons by diphtheria toxin and found that only NREM sleep was reduced. Varin et al. (2016) further found that even ablating only 30% of MCH neurons resulted in basal state and sleep deprivation following the decreased NREM sleep intensity. These results suggest that MCH neurons may have a dose-dependent effect in NREM sleep regulation.

In pathological states, functional abnormalities of MCH neurons may be associated with sleep disorders. Naganuma et al. (2018) found that in orexin-deficient mice, specific activation of MCH neurons by a chemical genetics technique (hM3Dq DREADD system) resulted in an imbalance in the regulation of REM sleep, manifested by somnolence and short latency to REM sleep transition (SLREM). This aberrant phenotype is fully reversed by the MCH receptor 1 antagonist SNAP 94847, suggesting that overactivation of MCH neurons in the context of orexin deficiency may lead to an abnormal invasion of REM sleep into the NREM sleep and wake periods.

Regarding the specific mechanism of sleep induction by MCH neurons, the current study revealed several possible pathways: (1) MCH neurons can directly inhibit orexin neuronal activity by releasing MCH neuropeptides (Rao et al., 2008; Apergis-Schoute et al., 2015); (2) some MCH neurons co-express GABA, and the GABAergic system promotes NREM sleep by inhibiting arousal and promoting network synergy. GABAergic tuberomammillary neurons in the ventrolateral preoptic nucleus (VLPO) directly inhibit orexin neurons in the hypothalamus and monoaminergic arousal centers (such as the locus coeruleus and nucleus), while MCH neurons can enhance this inhibition by releasing GABA or activating the VLPO pathway. This cascade inhibition of GABA, MCH, orexin forms a triple regulatory network, which jointly stabilizes the initiation and maintenance of NREM sleep (Elias et al., 2001; Del Cid-Pellitero and Jones, 2012) (Figure 1); (3) MCH neurons may influence the sleep-wake transition by modulating the release of other fast neurotransmitters (Chee et al., 2015); (4) recent studies have found that MCH neurons may increase NREM sleep and EEG δ-power by indirectly de-suppressing the reticular thalamic nucleus (RT) (Herrera et al., 2016). Together, these mechanisms constitute a complex network of MCH neurons regulating sleep, providing new perspectives for understanding the neural circuitry of sleep-wake regulation. At the same time, this sleep-promoting effect of MCH neurons is likely amplified by their inhibition of wake-promoting orexin neurons (Apergis-Schoute et al., 2015), illustrating a key node of the bidirectional inhibitory network.

### MCH is involved in hippocampus-dependent forgetting of memories during rapid eye movement sleep

MCH neurons are involved in memory regulation through the release of a variety of neurotransmitters (Chee et al., 2015), but the specific neurochemical mechanisms by which they mediate memory impairment have not been fully elucidated. It has been shown that cocaine-amphetamine-regulated transcription peptide (CART) may be specifically expressed in MCH neurons active during REM sleep (Cvetkovic et al., 2004; Hanriot et al., 2007). This finding is based on two key pieces of evidence: first, only MCH neurons co-localized with the CART project to the hippocampus; and second, CART-positive MCH neurons show specific activation during the REM sleep phase. In addition, the glutamatergic signaling pathway may be involved in the regulation of hippocampal function through a feed-forward inhibitory mechanism mediated by GABAergic interneurons (Chee et al., 2015). Notably, metabotropic glutamate receptors have been shown to be involved in active amnesia during sleep (Diering et al., 2017).

Shuntaro Izawa's team (Izawa et al., 2019) systematically elucidated the role of REM-active MCH neurons in hippocampus-dependent memory forgetting through a series of experiments. The study first confirmed the intensive projection of hypothalamic MCH neurons to the hippocampus. Subsequently, the researchers constructed the MCH-tTA; TetO Yellow Cameleon-Nano50 (YC) mouse model, which combined histochemical and electrophysiological methods to confirm that MCH neurons specifically express hM3Dq and are activated by CNO (clozapine-N-oxide).

At the behavioral level, the study assessed the effects of MCH neurons on memory using the Novel Object Recognition (NOR) and Contextual Fear Conditioning (CFC) tests. The results showed that (1) in the NOR test, administration of CNO after memory acquisition significantly impaired memory retention; (2) in the CFC test, MCH neuron activation significantly reduced freezing rate, suggesting impaired memory; and (3) inhibition of MCH neurons by AAV9-CAG-FLEX-hM4Di-mCherry viral vectors significantly improved NOR and CFC memory (Izawa et al., 2019); (4) long-term ablation of MCH neurons using diphtheria toxin A fragment (DTA) (Tsunematsu et al., 2014) consistently improves memory performance within 48 h. (5) Activation of MCH nerve endings in the hippocampus through optogenetic specificity significantly impairs memory function.

At the level of cellular mechanisms, whole-cell membrane clamp recordings showed that optogenetic activation of MCH nerve endings significantly reduced the firing frequency of hippocampal CA1 pyramidal neurons while increasing the frequency and amplitude of inhibitory postsynaptic currents (IPSCs), suggesting enhanced GABAergic inhibitory input. To further resolve the heterogeneity of MCH neurons, the investigators labeled MCH neurons by AAV9-CMV-FLEX-GCaMP6f viral vector and recorded calcium signals at the single-cell level. The results showed that of the 146 recorded cells, 34.9% were active during wakefulness, 52.8% were active during REM sleep, and 12.3% were active in both states, and these subpopulations were randomly distributed in the LHA. The presence of these subpopulations suggested the possibility of different roles for wake-active and REM-active MCH neurons in memory. Finally, state-dependent optogenetic inhibition experiments revealed that inhibition of MCH neurons only during REM sleep significantly improved memory, whereas inhibition during wakefulness or NREM sleep had no such effect. This finding of Izawa et al., combined with the experimental conclusions they obtained above, confirms each other, proving the specific role of MCH neurons active in the REM sleep stage in memory impairment. This active forgetting process during REM sleep, mediated by MCH neurons, stands in contrast to the wake-promoting and memory-encoding role of orexin neurons, demonstrating their antagonistic functions within the memory dimension of the network. At the same time, in order to determine whether the activation of MCH neurons is a necessary condition for REM amnesia, it is necessary to design experiments in the future to prevent its REM activation (such as specific knockout of ion channels related to the REM activity of MCH neurons) and observe whether the amnesia process is blocked.

### MCH neurons can modulate hippocampus-dependent memory formation by regulating hippocampal synaptic plasticity thresholds

Synaptic plasticity in the hippocampus is thought to be the cellular basis of memory, but this plastic change does not occur in isolation. The hippocampus receives multiple inputs and projections from multiple brain regions, which allows it to modulate synaptic plasticity during memory based on unique environmental conditions. Melanin-concentrating hormone (MCH) neurons in the hypothalamus are one of these important sources of input.

MCH neurons originate in the lateral hypothalamus (LH) and project widely throughout the brain, especially in densely innervated areas of the hippocampus (Harris and Burdakov, 2024). Inhibition of MCH neuron activity during object recognition negatively affects memory formation (Kosse and Burdakov, 2019). *In vitro* studies have shown that exogenous application of MCH (4 and 11 μM) can lead to a long-term increase in the efficacy of hippocampal synaptic transmission (Varas et al., 2002), while when MCH neurons or their receptors are genetically deleted, the plasticity of the hippocampus is impaired, requiring stronger stimulation to induce long-term potentiation (LTP). This gene deletion increases the induction threshold of LTP by affecting hippocampal plasticity (Pachoud et al., 2010; Le Barillier et al., 2015).

Based on these findings, Harris and Burdakov (2024) proposed the hypothesis that MCH input may modulate synaptic plasticity in the hippocampus and that increasing MCH input should help promote this plasticity. To test this hypothesis, they activated MCH axons in hippocampal slices by optogenetic techniques. Three classical plasticity induction protocols were used in the study: weakly enhancing stimuli (one tonic stimulus, usually triggering a brief post-tonic potentiation, PTP), strongly enhancing stimuli (four tonic stimuli, usually triggering a long-term potentiation, LTP), and a single inhibitory stimulus (900 single pulses at 1 Hz, usually triggering a long-term inhibition, LTD). These stimuli were alternated with blue light stimulation of MCH axons to investigate the effect of MCH axon activation on cone cell excitatory field potentials (fEPSP). The results showed that activation of MCH axons had no significant effect on the persistent enhancement or inhibition induced by strong electricity, but could shift the effect of weak stimulation from transient to persistent enhancement. The final conclusion is that activation of MCH axons in the hippocampus by optogenetics can lower the threshold of persistent enhancement induced by electrical stimulation, thereby promoting synaptic plasticity in the hippocampus.

In summary, MCH in the hippocampus can make the induction of long-term potentiation (LTP) easier by lowering the threshold of synaptic plasticity. It has been shown that LTP in hippocampal synapses is positively correlated with hippocampus-dependent memories (Wang et al., 1997), thus, MCH neurons play a key role in regulating hippocampal synaptic plasticity and the formation of hippocampus-dependent memories.

## Orexin neurons are involved in arousal and REM and modulate situational and fear memory

### Orexin neurons inhibit sleep to promote arousal and maintain the arousal state

Multiple nuclei in the brain that regulate sleep-wake balance receive projections from lateral hypothalamic orexin neurons, including the locus coeruleus (LC) (Soya et al., 2017), dorsal raphe nucleus (DRN) (Yang et al., 2019), tuberomammillary nucleus (TMN) (Bayer et al., 2001), pedunculopontine tegmental nucleus/laterodorsal tegmental nucleus (PPT/LDT) (Ishibashi et al., 2015), and basal forebrain (BF) (Zhang et al., 2016) (Figure 2). Thus, orexin neurons have a key role in stabilizing and maintaining the arousal state (summarized in Table 2). For example, individuals with narcolepsy exhibit excessive daytime sleepiness, sleep episodes, and unstable arousal due to the absence of orexin neurons. Animal models of orexin deficiency almost completely recapitulate this narcolepsy phenotype (Chemelli et al., 1999; Mochizuki et al., 2004; Branch et al., 2016).

**Table 2:** Summary of studies that investigated the role of the orexin system in sleep-wake regulation.

**Experiment**	**Effect on arousal**	**References**
Orexin gene knockout	Increased in the number of NREM and REM bouts during the dark phase Decreased in the duration of NREM and REM bouts during the dark phase Decreased in REM latency during the dark phase Decreased in the duration of wake bouts during the dark phase Alterations in the circadian frequencies of REM episodes Increased fragmentation of the sleep-wake cycle Hypersomnia	Chemelli et al., 1999; Willie et al., 2003; Mochizuki et al., 2004
Orexin neurons ablation	Increased in REM amount during the dark phase Increased in the duration of REM bouts during the dark phase Decreased in the duration of wake bouts during the dark phase Increased fragmentation of the sleep-wake cycle	Hara et al., 2001
OX2-receptor deletion	Increased fragmentation of the sleep-wake cycle Decreased in the duration of wake during the dark phase Decreased in the duration of NREM during the dark phase Decreased in REM latency during the dark phase	Willie et al., 2003; Mochizuki et al., 2004
Optogenetic stimulation of orexin neurons	Increased the transition to wake from NREM or REM 5–30 Hz light pulse trains decreased wake latency Strong reduction of REM duration	Adamantidis et al., 2007
Chemo activation of orexin neurons	Increased wake amounts during the light phase Decreased in NREM amounts during the light phase Decreased in REM amounts during the light phase Modest increase in wake amounts during the dark phase Increased in the latency from wake to REM during the dark phase	Sasaki et al., 2011

Studies have shown that orexin neurons enhance the waking state by activating other arousal centers, such as locus coeruleus noradrenergic neurons (Carter et al., 2012). Humans and animals lacking orexin neurons typically experience daytime somnolence, sleep attacks, and an unstable waking state. Narcolepsy is thought to be a clinical manifestation of dysfunctional central orexin signaling, and numerous studies have confirmed that type I narcolepsy results from deficient orexin signaling caused by loss of orexin neurons (Overeem et al., 2001; Lin et al., 2002; Kornum et al., 2011). The strong correlation between narcolepsy and the human leukocyte antigens DRD2 or DQB1^*^0602 suggests that orexin neurons may be subjected to autoimmune destruction (Mignot, 2004; Fontana et al., 2010; Kornum et al., 2011; Singh et al., 2013; De la Herrán-Arita and García-García, 2014). A study by Roberto De Luca et al. utilized *in vivo* and *ex vivo* techniques such as optogenetics, neural circuit mapping, and single-cell transcriptional analysis to show that orexin neurons promote arousal by indirectly inhibiting neurons in the ventral lateral preoptic nucleus (VLPO) (De Luca et al., 2022). The VLPO is an important region for initiating and maintaining sleep and contains neurons that are active during sleep, and damage to it can lead to severe and persistent insomnia (Sherin et al., 1996; Ma et al., 2019). Studies have identified a functional polysynaptic circuit between orexin neurons and sleep-promoting VLPO GABA/Gal neurons, and by activating this circuit, orexin neurons can “turn off” VLPO GABA/Gal neurons, thereby promoting arousal. Orexin deficiency can lead to a decrease in excitability of VLPO GABA neurons, reducing feedforward inhibition of VLPO GABA/Gal neurons, thereby affecting sleep status. In the study by Vittoz and Berridge (2006), it was found that the increase in PFC (Prefrontal cortical) DA (Dopamine) levels induced by orexin was significantly positively correlated with the duration of wakefulness (*r* = 0.61), and the amplitude of DA release far exceeded the baseline fluctuation caused by wakefulness alone (about 25% vs. 60–80%). In the experiment, they injected orexin-1 into the VTA (ventral tegmental area) to activate orexin receptors and found that the activity of PFC-projecting DA neurons was selectively enhanced, PFC DA release increased, and the cortical network was activated through D1/D2 receptors, promoting wakefulness and coordinating high-arousal- related behaviors. In *in vitro* slice electrophysiology studies, orexin A and orexin B were found to increase the rate of issuance of monoaminergic neurons in LC (Liu et al., 2002; Kargar et al., 2018), DRN (Liu et al., 2002; Ishibashi et al., 2016), and TMN (Bayer et al., 2001; Eriksson et al., 2001), and cholinergic neurons in BF and LDT (Eggermann et al., 2001; Burlet et al., 2002). In optogenetic experiments, activation of LC neurons induced the transition from sleep to wakefulness (Carter et al., 2012), whereas activation of orexin neurons enhanced neuronal activity in the LC (Soya et al., 2017). These findings suggest that orexin neurons maintain and consolidate the arousal state by modulating monoaminergic and cholinergic neuronal activity in downstream nuclei.

The clinical regulation of the orexin pathway has two-way therapeutic potential: orexin receptor agonists (such as TAK-994) improve the maintenance of arousal in narcolepsy by compensating for endogenous orexin deficiency (Mahoney et al., 2019), while antagonists (such as suvorexant) treat insomnia by blocking arousal signals (Herring et al., 2012). This two-way regulation highlights the core role of the orexin system in sleep-wake balance, but its potential impact on memory networks (such as MCH- dependent REM memory forgetting) needs to be vigilant (Izawa et al., 2019).

### Orexin neurons inhibit REM sleep and mediate regulation of rapid eye movement sleep stabilization

A stable state of alertness is essential for the maintenance of a wide range of critical brain functions, a process that relies on the regulation of various neurochemical signals, among which the orexin neurons of the hypothalamus play an indispensable role (Chemelli et al., 1999; Hara et al., 2001; Mahler et al., 2014). Orexin neuron signals are conveyed through widely distributed fibers and distributed to different brain circuits to coordinate the sleep-wake state (Peyron et al., 1998; Pintwala and Peever, 2017). These neurons are interconnected with arousal-promoting brain regions such as the tuberomammillary nucleus, the locus coeruleus, and the dorsal raphe nucleus (Peyron et al., 1998).

Studies have shown that orexin neurons inhibit REM sleep by acting on these brain regions. Early studies using acute orexin neuron activation reported robust REM suppression (Adamantidis et al., 2007; Sasaki et al., 2011), suggesting an inhibitory role in REM initiation. However, Feng et al. revealed a nuanced function: orexin input to the SLD (sublaterodorsal tegmental nucleus) during ongoing REM enhances neuronal synchrony and prolongs REM episodes (Feng et al., 2020, 2024). This apparent contradiction is resolved when considering temporal context: Orexin neurons play a biphasic regulatory role in REM sleep through their projections, they inhibit the initiation of REM sleep during arousal. During REM sleep, they are involved in maintaining the stability and integrity of REM sleep fragments. In the study of Sasaki et al. (2011), the activity of orexin neurons was specifically inhibited by DREADD (Designer Receptors Exclusively Activated by Designer Drugs) technology. The results showed that in the bright phase (mouse rest period): the awakening time was significantly reduced, and the total amount of NREM and REM sleep increased, indicating that orexin neurons play a key role in maintaining daytime wakefulness. In the dark phase (mouse activity phase): the awakening time was further reduced, the amount of NREM and REM sleep continued to increase, and the latency of the transition from wakefulness to REM sleep was shortened, suggesting that the inhibition of the orexin system will weaken the awakening homeostasis and accelerate the start of REM sleep. On the other hand, a study by Burgess et al. (2013) found that a reduction in orexin disinhibits REM sleep-related regions, such as neurons in the sub-LDT and LDT/PPT, thereby promoting REM sleep. Together, these studies confirm the inhibitory effect of orexin neurons on REM sleep.

Effective homeostatic regulation of REM sleep is critical for maintaining a variety of important functions such as emotional processing, motor control, and energy homeostasis (Liu et al., 2002; Ishibashi et al., 2016). It has been reported that disturbances in REM sleep homeostasis may lead to related clinical disorders such as depression, attention deficit hyperactivity disorder (ADHD), and obesity (Wang et al., 2015; Bassetti et al., 2019; Daiber et al., 2020; Tan et al., 2022). Recent studies have observed that orexin deficiency leads to impaired REM sleep quality (Knudsen et al., 2010; Bastianini et al., 2012). The sublaterodorsal tegmental nucleus (SLD) is considered to be an essential region for the generation and maintenance of REM sleep, and it integrates a variety of neurochemical signals during the sleep-wake cycle (Knudsen et al., 2010; Bastianini et al., 2012). Feng et al. (2020) found that a portion of orexin neurons projected to the SLD and exhibited activation during REM sleep. In the SLD, orexin directly stimulates orexin receptor-positive neurons and increases gap junction conductance between neurons. Their interaction spreads the orexin-induced excitation, which activates the entire SLD network. The activated SLD network exhibited an increase in the probability of synchronized firing, facilitating the connection between the SLD and its downstream targets to enhance the output of the SLD. Using optogenetics and fiber photometry, the team found that orexin-enhanced SLD output prolonged REM sleep by consolidating the activation state/dystonic inhibition state of the brain state. A 17% reduction in the amount of REM sleep was observed following silencing of orexin signaling in the SLD, accompanied by dystonic disruption. These findings reveal a stabilizing role for orexin in REM sleep. In a recent study by Feng et al. (2024) the REM sleep-related neuronal dynamics of orexin neurons in the SLD and other projection-labeled orexin neurons during the sleep/wake cycle were observed by fiber-optic photometry and correlated with different physiological REM sleep parameters, and there is a significant positive correlation between the activation level of orexin neurons in SLD and the duration of the REM sleep interval. This suggests that orexin neurons play a crucial role in the sleep/wake cycle for the SLD neural pathway in relieving REM sleep stress. The stabilizing role of orexin on REM sleep may involve counteracting potential over-excitation within REM-generating circuits, which could be influenced by MCH neuronal activity, highlighting the network-level coordination required for REM sleep integrity.

### Orexin system and memory regulation

Orexin neurons dynamically regulate memory encoding, consolidation, and retrieval processes through multi-level molecular and loop mechanisms, and their functions are highly dependent on the body's energy metabolism status. At the level of synaptic plasticity, Selbach et al. (2010) first revealed that orexin-A enhances NMDA receptor-mediated calcium influx by activating the OX1 receptor (OX1R) in the hippocampal CA1 region (Selbach et al., 2010), thereby triggering a cascade reaction of ERK/MAPK and PI3K/Akt signaling pathways, promoting the formation of long-term potentiation (LTP). This mechanism was further validated in Zhao et al.'s (2014) experiment, where they found that OX1R agonists can specifically enhance hippocampal LTP and improve spatial memory deficits in aging model mice. Behavioral studies have shown that injecting orexin-A into the lateral ventricle can significantly improve spatial memory deficits in aging or chronic stress model mice (Telegdy and Adamik, 2002), while selective OX1R antagonists (such as SB-334867) can block the consolidation process of fear memory (Akbari et al., 2006).

It is worth noting that orexin has a significant metabolic dependence on memory regulation: Vittoz and Berridge (2006) confirmed through microdialysis that under acute starvation, the activity of orexin neurons in the hypothalamus increases, enhance the release of dopamine from the ventral tegmental area (VTA) to the prefrontal cortex. This pathway is directly related to the preferential encoding of foraging-related environmental memory. Meanwhile, Yang et al. (2013) demonstrated through optogenetic techniques that activating orexin neurons can significantly enhance the theta rhythm synchronization of the amygdala-hippocampus loop, thereby promoting the rapid consolidation of fear memories. In cases of chronic energy surplus, such as that induced by a high-fat diet, a series of experiments conducted by Forte et al. (2021) demonstrated that the downregulation of orexin signaling results in reduced hippocampal neurogenesis, an increased synaptic plasticity threshold, and impaired pattern separation ability. Conversely, exogenous supplementation with orexin-A can partially reverse this damage by restoring the endogenous cannabinoid 2-AG-CB1R signaling pathway (Forte et al., 2021). Leptin and ghrelin regulate orexin activity via antagonistic mechanisms, which can suppress or enhance memory. Metabolic abnormalities, such as hyperglycemia or hypoglycemia, alter memory encoding efficiency through oxidative stress or the AMPK [adenosine 5′-monophosphate (AMP)-activated protein kinase] pathway, revealing the multi-scale plasticity regulation of metabolic signals on memory.

The regulatory mechanism of metabolic hormones has also been extensively studied: Yamanaka et al. (2003) found that leptin inhibits orexin expression by activating the JAK2-STAT3 pathway, which is closely related to the decrease in hippocampal BDNF (brain-derived neurotrophic factor) levels and spatial memory impairment (Paz-Filho et al., 2008). On the contrary, the groundbreaking study by Diano et al. (2006) showed that gastric-derived ghrelin directly activates orexin neurons through its receptor GHSR1a, enhancing long-term consolidation of amygdala- dependent fear memory. Under pathological conditions, the diabetes model of Han et al. (2020) shows that hyperglycemia inhibits the membrane localization of hippocampal OX1R through oxidative stress, leading to impairment of long-term memory retrieval; the *in vitro* electrophysiological experiment by Burdakov et al. (2005) revealed that acute hypoglycemia temporarily enhances hippocampal episodic memory encoding efficiency through AMPK-dependent synaptic remodeling. Orexin dynamically regulates memory through metabolic state: acute hunger activates the hypothalamus-VTA dopamine pathway to strengthen foraging memory, while chronic energy surplus impairs hippocampal plasticity; its dual roles (rapid neurotransmitter release and long-term synaptic remodeling) form the core hub of metabolism-memory interaction.

Clinical studies have shown that the orexin system, particularly orexin-A, plays a critical regulatory role in fear-related disorders. Plasma orexin-A levels are significantly elevated in patients with panic disorder, while those with PTSD exhibits a decreasing trend (Johnson et al., 2010; Strawn et al., 2010). Additionally, narcolepsy patients may experience deficits in fear memory acquisition due to orexin deficiency (Ponz et al., 2010; Shi et al., 2018). Pavlovian fear conditioning experiments found that orexin-A levels are negatively correlated with the extinction of recent fear memories—individuals with high orexin-A levels maintain lower fear responses after fear extinction, though this does not significantly affect the extinction of remote fear memories, suggesting differences in regulatory mechanisms across memory stages. The underlying mechanisms may involve orexin-A enhancing the firing frequency of CA1 neurons and promoting dentate gyrus neurogenesis via the OX1 receptor (OX1R) (Zhao et al., 2014; Chen et al., 2017). Furthermore, orexin-A injections can activate hippocampal plasticity kinases (e.g., ERK) to enhance long-term potentiation (LTP), thereby consolidating memory (Wayner et al., 2004; Myskiw et al., 2014). In PTSD animal models, stressed rats (SPS model) exhibit hippocampal volume reduction, decreased hypothalamic orexin-A levels, and upregulated hippocampal OX1R/OX2R expression. However, lateral ventricle injections of orexin-A can partially reverse spatial memory deficits and appetite disorders (Han et al., 2020). These findings suggest that the orexin system, by dynamically regulating hippocampal plasticity and fear memory extinction, may serve as a potential intervention target for Post-Traumatic Stress Disorder (PTSD) and other conditions. However, the differential regulation of recent and remote memories requires further investigation (Fullana et al., 2018; Krystal et al., 2019).

The orexin system also regulates learning and memory by influencing the amygdala (BLA). Multiple neurotransmitter systems within the BLA are involved in the regulation of hippocampal synaptic plasticity, including long-term potentiation (LTP) and long-term depression (LTD). In studies employing the LTP model, it has been reported that blocking orexin correlates with inhibition of LTP in the perforant pathway-dentate gyrus. This suggests that the blockade of the orexin system may modulate hippocampal function via the BLA, thereby impairing memory consolidation and inhibitory avoidance learning. This further emphasizes the importance of the orexin system in learning and memory (Ardeshiri et al., 2018; Noorani et al., 2021). These pieces of evidence indicate that the orexin system is not only a core hub for the interaction between energy states and memory functions, but its activity changes can also dynamically adjust the allocation strategy of memory resources to prioritize the storage and retrieval of survival-related information.

### Reciprocal interactions between orexin and MCH neurons: a proposed bidirectional network framework

Orexin and melanin-concentrating hormone (MCH) neurons within the lateral hypothalamus exhibit anatomical proximity and functional interactions consistent with a *proposed* bidirectional inhibitory network model. This model offers a valuable framework for conceptualizing their coordinated roles in sleep-wake regulation and learning-forgetting balance, integrating findings from numerous indirect studies. Anatomically, Orx and MCH neurons show significant spatial co-localization (Trivedi et al., 1998; Hervieu et al., 2000; Kilduff and de Lecea, 2001; Marcus et al., 2001; Saito et al., 2001) and project to overlapping arousal, sleep, and memory centers (e.g., LC, DRN, hippocampus, SLD, Figures 1, 2). Crucially, *in vitro* and acute manipulation studies suggest reciprocal inhibition: optogenetic activation of Orx neurons suppresses MCH activity via local GABAergic interneurons (Apergis-Schoute et al., 2015), while MCH neuropeptides directly inhibit Orx neuronal firing (Rao et al., 2008). This forms a plausible *electrophysiological basis* for bidirectional crosstalk within this proposed network.

Within the sleep-wake dimension, the activities of these neuronal populations often appear antagonistic and complementary: Orx neurons stabilize wakefulness by activating monoaminergic/cholinergic systems, while MCH neurons promote NREM and REM sleep by inhibiting arousal nuclei and potentiating sleep centers. Their interplay in REM sleep control is particularly nuanced. Orx neuronal activity primarily inhibits REM onset and may stabilize ongoing REM architecture (Adamantidis et al., 2007; Feng et al., 2020), whereas MCH neurons potently facilitate REM generation (Jego et al., 2013). The pathological observation that loss of Orx inhibition (e.g., in narcolepsy models) leads to MCH-driven REM dysregulation (Naganuma et al., 2018) is *consistent with* the network model predicting instability when inhibitory balance is disrupted.

Memory regulation reveals complementary state-dependent specializations aligning with the network concept. During wakefulness, Orx dominance enhances encoding: it potentiates hippocampal LTP via OX1R signaling (Selbach et al., 2010) and synchronizes amygdala-prefrontal theta for fear consolidation (Yang et al., 2013). Conversely, during REM sleep, MCH neuronal activity promotes active forgetting: its release in the hippocampus enhances GABAergic inhibition of CA1 neurons, impairing memory retention (Izawa et al., 2019). This temporal division—Orx strengthening salient traces in wake, MCH weakening less relevant ones in REM—suggests a *potential* bidirectional mechanism for cognitive efficiency.

The severe phenotype observed in mice with dual ablation of both neuron populations (OREXINMC mice, Hung et al., 2020), including exacerbated cataplexy, reduced REM, and a novel pathological “DT sleep” state, highlights functional interdependence and suggests that MCH neurons act as crucial stabilizers when Orx signaling fails. This phenotypic collapse supports the concept of network-level dysfunction but does not definitively prove the physiological existence of a *functional unit* under normal conditions.

However, it is critical to emphasize that the bidirectional network model, while integrating substantial indirect evidence, remains a theoretical framework; its physiological existence still needs further research to verify.

## Discussion

The dynamic interplay between orexin and melanin-concentrating hormone (MCH) neurons extends beyond their individual roles, forming the basis for a *compelling theoretical model* of a functionally integrated bidirectional network within the lateral hypothalamus. Our synthesis demonstrates that reciprocal inhibition—supported by *in vitro* and acute manipulation studies showing Orx activation suppresses MCH neurons via GABAergic interneurons (Apergis-Schoute et al., 2015), and MCH release inhibits Orx firing (Rao et al., 2008)—provides a plausible *core circuitry* for this proposed network. This foundational interaction underpins a model of *state-dependent complementarity*: Orx stabilizing wakefulness and prioritizing salient memory encoding, while MCH promoting sleep (particularly REM) and facilitating hippocampal-dependent forgetting. The bidirectional framework effectively *resolves apparent contradictions* in the literature, such as Orx's dual role in REM sleep (inhibiting onset while stabilizing episodes; Adamantidis et al., 2007; Feng et al., 2020), by framing them as context-dependent modulations within the network logic. Similarly, variations in MCH's effects on NREM sleep (Tsunematsu et al., 2014 vs. Konadhode et al., 2013) likely stem from methodological differences (acute vs. chronic manipulation) or compensatory mechanisms, highlighting the system's complexity and plasticity.

The severe phenotype of dual Orx/MCH-ablated mice (OREXIN^∧^MC^∧^, Hung et al., 2020) exemplifies *network-level collapse*, suggesting functional interdependence. Exacerbated cataplexy, reduced REM, and emergent “DT sleep” indicate that MCH neurons are not merely REM promoters but may act as stabilizers counteracting pathological state transitions when Orx signaling fails. This underscores *emergent properties* arising from their interaction. Disruptions within this proposed network cascade: Orx deficiency destabilizes circadian arousal, permitting MCH-driven REM dysregulation (Naganuma et al., 2018), which could subsequently impair sleep-dependent memory filtering (Izawa et al., 2019). This cascade—from circadian disintegration to synaptic maladaptation—provides a *mechanistic hypothesis* for comorbidities in disorders like narcolepsy (sleep attacks, cataplexy, emotional memory deficits) and PTSD (REM fragmentation, intrusive memories).

Clinically, this bidirectional network model provides a *unifying conceptual framework* for intervention. Targeting a single node (e.g., orexin agonists for narcolepsy; MCH antagonists for PTSD forgetting) may yield benefits but risks unintended network imbalances. For instance, while Orx antagonists improve insomnia (Herring et al., 2012), their long-term impact on MCH-dependent memory pruning requires careful evaluation. Future therapeutic strategies could benefit from considering *simultaneous modulation of network equilibrium*—e.g., combining Orx stabilization with MCH circuit modulation to ameliorate both sleep and memory symptoms in trauma-related disorders.

Crucially, significant gaps remain in validating this model as the *physiological reality* governing sleep-wake and memory states. Future research must explicitly address: 1. The Physiological Reality of the Network: Direct evidence demonstrating that Orx and MCH neurons function as a reciprocally inhibitory, oscillatory unit *in vivo* during natural behavior is paramount. This requires techniques capable of resolving real-time, bidirectional interactions between these specific neuronal populations across sleep-wake states (e.g., dual-color fiber photometry, multi-unit recordings with optogenetic tagging, or advanced *in vivo* imaging). 2. Molecular Mechanisms of Crosstalk: The specific receptors and intracellular signaling pathways mediating the reciprocal inhibition, particularly MCH's effect on Orx neurons, need definitive identification. Research must determine if these mechanisms are static or dynamically modulated by factors like circadian phase, metabolic state, or prior sleep history. 3. Memory Dynamics within the Network: How do the opposing memory functions (Orx-driven encoding vs. MCH-driven forgetting) interact dynamically at the synaptic and circuit level? Does disruption of REM sleep or MCH activity directly impair the *efficacy* of Orx-dependent encoding during subsequent wakefulness, or vice versa? What are the precise hippocampal (and extra-hippocampal) circuit mechanisms mediating the “forgetting” signal during REM sleep? Studies combining state-specific manipulations with detailed behavioral memory assays and circuit mapping are essential. 4. Metabolic and Circadian Modulation: How do metabolic signals (leptin, ghrelin, glucose) and circadian inputs dynamically tune the gain and interaction within the Orx-MCH network? Understanding this integration is vital for explaining state-dependent vulnerabilities and comorbidities like obesity-related sleep and memory disturbances. 5. Subpopulation Specificity: Does functional heterogeneity within Orx and MCH neurons (e.g., REM-active vs. wake-active MCH subsets; Izawa et al., 2019; Orx neurons projecting to SLD vs. LC) fine-tune network output in specific ways? Resolving subpopulation dynamics will be key for precision targeting.

In conclusion, while the Orx-MCH bidirectional network model offers a powerful and integrative framework for understanding the coordination of arousal, sleep architecture, and memory homeostasis, it remains a theory awaiting full physiological validation. The model compellingly suggests that disruption of this integrated hub propagates dysregulation from circadian/sleep instability to mnemonic dysfunction, providing a testable hypothesis for neuropsychiatric comorbidities. Addressing the outlined research priorities—particularly establishing the *in vivo* dynamics and molecular specifics of the reciprocal interactions—will be essential to transform this valuable perspective into a firmly established physiological principle and guide the development of effective, multi-targeted therapeutic strategies.

## Data Availability

The data resource and materials are available from the corresponding author upon reasonable request.
